# A Systematic Comparative Study on the Diagnostic Value of Transabdominal Ultrasound in Patients with Pancreatic Cystic Lesions

**DOI:** 10.3390/jcm11206176

**Published:** 2022-10-19

**Authors:** Viktoria Hentschel, Jennice Beckmann, Wolfgang Kratzer, Frank Arnold, Thomas Seufferlein, Benjamin Walter, Alexander Kleger, Martin Müller

**Affiliations:** 1 Department of Gastroenterology, Clinic of Internal Medicine 1, University Hospital of Ulm, 89081 Ulm, Germany; 2 Institute for Molecular Oncology and Stem Cell Biology, University Hospital of Ulm, 89081 Ulm, Germany

**Keywords:** pancreatic cyst, ultrasound imaging, pancreatic intraductal neoplasms

## Abstract

Pancreatic cystic lesions are a frequent incidental finding in abdominal imaging. Despite its usually benign background, a small fraction exhibiting features suspicious for cancerous development demands continuous follow-up or surgical removal. Current guidelines advocate magnetic resonance imaging and endoscopic ultrasound to evaluate the risk of malignancy, whereas transabdominal ultrasound is perceived as subordinate imaging. The objective of this study was to analyze cyst detection rates of latest-generation ultrasound machines compared to magnetic resonance imaging, computed tomography, and endosonographic ultrasound and to determine inter-rater reliability. The results showed that large cysts facilitate their visualization by transabdominal ultrasound while detection rates are independent of the anatomical part of the pancreas in which they were sited. Changes in the pancreatic duct width, a connection to the pancreatic duct system, and the architectural characteristics of cysts are poorly recognized by transabdominal ultrasound compared to magnetic resonance imaging and endoscopic ultrasound. Computed tomography imaging is preferred over transabdominal ultrasound to detect calcifications and regional lymphadenopathy. Even if conducted by experienced investigators, transabdominal ultrasound examinations fail to agree with magnetic resonance imaging scans regarding cyst detection rates (κ = 0.093).

## 1. Introduction

Pancreatic cysts constitute a heterogenous group of lesions that physicians often become aware of during transabdominal ultrasound (TAUS) examinations or abdominal cross-sectional imaging conducted for unrelated reasons. Such incidental findings are present in 1.2–2.6% of patients evaluated with abdominal computed tomography (CT) scan and up to 13.5% of patients undergoing magnetic resonance imaging (MRI) [1,2,3]. The prevalence of pancreatic cystic lesions (PCLs) is age-dependent and affects approximately 8–10% of individuals older than 70 years [4,5]. PCLs may either have neoplastic or non-neoplastic origins, with the latter being almost exclusively attributable to inflammatory fluid collections [6]. Statistically, 8% of pancreatic malignancies are thought to arise from one of the two precursor PCLs, intraductal papillary mucinous neoplasm (IPMN), and mucinous cystic neoplasm (MCN) [7,8]. The chances of an incidental PCL being a mucinous invasive neoplasm or ductal adenocarcinoma have been estimated to be 10 and 17 in 100,000, respectively [9]. This underscores the need for workable tools to distinguish between PCLs that are safe to watch and those requiring timely resection, while at the same time preventing overdiagnosis and unnecessary interventions. The European Study Group on Cystic Tumours of the Pancreas and the Clinical Guidelines Committee of the American Gastroenterology Association unanimously recommend MRI and endoscopic ultrasound (EUS) as first-line imaging options to characterize the morphology of a given PCL and to draw inferences about their presumable risk of malignancy [9,10]. Residual diagnostic uncertainties are reduced to an acceptable level by fine-needle aspiration of cyst fluids for cytological, tumor markers, and molecular analysis. On the contrary, TAUS continues to play a negligible role in those guidelines for a number of reasons. Foremost, a high dependency of TAUS on the individual investigator’s expertise may be substantially aggravated by patient-related factors such as gas bloating, abdominal distention, and obesity. As a result, only a few studies have addressed how precisely PCLs of a specific size range, complexity, and localization can be delineated by TAUS compared to standard pancreatic imaging.

In light of sparse data availability on the utility of modern TAUS technology, we felt encouraged to provide a comprehensive retrospective study comparing the diagnostic performance of TAUS versus EUS, MRI, und CT with respect to a variety of PCL-defining features. In addition, we investigate the inter-rater reliability between TAUS and MRI and how it is influenced by the investigators’ practical proficiencies. Lastly, we discuss the potential strengths of TAUS and how they could be profitably exploited for an efficient management of PCLs.

## 2. Materials and Methods

### 2.1. Enrollment of Study Population

The present retrospective single-center study was conducted at Ulm University Hospital, Germany. After obtaining approval by the institutional ethic committee, the clinic’s electronical records were systematically searched for patients undergoing out-patient care for pancreatic diseases. The query yielded a total of 613 patients with documented visits at our specialized outpatient clinic during the time period of January 2014–May 2020. Out of those, 258 patients encoded with the diagnosis of a PCL were enrolled for further analysis. Cystic lesions of interest encompassed IPMN (main duct type, md-IPMN; branch duct type, bd-IPMN; and mixed type, m-IPMN), MCN, serous cystic neoplasm (SCN), walled-off necrosis (WON), post-pancreatitis fluid collections (PFC), and PCLs termed as “not further classified”. Patients (1) without a confirmatory TAUS scan of an incidental PCL; (2) in whom initial diagnostic work-up had not been complemented by at least one additional imaging modality such as EUS, MRI, and CT; (3) who had been diagnosed with a PCL before January 2014; and (4) with a time span extending beyond 6 months between two different imaging studies were excluded, resulting in a final study population of 147 patients. In addition to pancreas-specific imaging studies, the following aspects pertinent to the patients’ medical history and laboratory findings were compiled: (1) smoking, (2) alcohol abuse, (3) epigastric discomfort, (4) endocrine insufficiency (defined by decreased C-peptide levels and overt diabetes mellitus requiring exogenous insulin supplementation), and (5) exocrine insufficiency (defined by pancreatic elastase 1 < 50 µg/g stool).

### 2.2. Imaging Studies

All 147 study subjects initially underwent TAUS to examine the pancreas. Out of those, 107 examinations were conducted at Ulm University Hospital. Another 40 examinations were performed by licensed health care providers such as general practitioners and specialists. Investigators were categorized into two groups: hospital staff certified to have performed more than 30,000 examinations were considered (1) ultrasound experts while any external investigators or those falling below the minimum number of examinations were subsumed under (2) basic-level trained ultrasonographers. However, all investigators were qualified to perform TAUS examinations independently, and no additional re-survey by an ultrasound expert was required. All investigators performing EUS were specialists with long-term expertise and skills in the field. Cross-sectional imaging modalities such as MRI/magnetic resonance cholangiopancreatography (MRCP), and CT were provided both by the hospital’s own radiology department and other health care facilities. As opposed to the basic TAUS, the extent of further diagnostic work-up was variable among patients, usually not covering the complete series of available and appropriate imaging modalities. Suspicious TAUS findings were assessed by EUS in 129 patients, out of which 114 were performed in the clinic. Seventy-nine patients were followed up with MRI (university hospital clinic: 45 patients, external health care providers: 34 patients). Eighty-one patients were evaluated with a CT scan (university hospital clinic: 42 patients, external health care providers: 39). Based on the findings obtained from different imaging studies, all patients were ultimately referred to the hospital’s outpatient clinic specialized in pancreatic diseases for consultation, treatment, and long-term follow-up.

### 2.3. Data Acquisition

All PCLs were characterized according to a predefined set of sonographic features:

- Type of cyst: IPMN, SCN, MCN, PFC, WON;
- Number of cysts: 0, 1, 2–3, >3;
- Location of cyst: head, body, tail of pancreas, uncinate process;
- Size: maximum diameter of largest cyst;
- Presence of calcifications: intraparenchymal, intracystic;
- Configuration of cyst: monocystic, polycystic;
- Internal structure of cyst: solid contents, nodules, septum/wall thickening, hemorrhage;
- Connection to major duct, side duct, or both (md-IPMN, bd-IPMN, and m-IPMN, respectively);
- Dilation of pancreatic major duct >5 mm, segmental changes in width;
- Dilation of common bile duct >7 mm;
- Compression of neighboring organs (vessels, stomach, duodenum, kidney, spleen);
- Enlargement of regional lymph nodes.

### 2.4. Ultrasound Units

The following ultrasound units were used for examinations performed at university hospital Ulm:

- GE Logiq E9 (year launched: 2008–2015; CE marking: 0459);
- Siemens Acusion S3000 (year launched: 2012; CE marking: 0123);
- Toshiba Aplio 500 (year launched: 2011; CE marking: 0197);
- Toshiba Aplio i800 (year launched: 2016; CE marking: 0197);
- Mindray TE5 und TE7 (year launched: 2020; CE marking: 0123);
- Philips IU22CE (year launched: 2004; CE marking: 0086);
- Philips Epiq 7 (year launched: 2013; CE marking: 0086);
- Hitachi Ascendus (year launched 2011; CE marking: 0123).

### 2.5. Statistical Analysis

All data were organized in Microsoft Excel and converted into a scalable format to allow computation of the mean, standard deviation, minimum, maximum, and relative quantities. Subsequently, the findings from TAUS were correlated with advanced imaging methods such as EUS and MRI/CT and with a definitive diagnosis, as concluded in the discharge summary. Specifically, we measured the sensitivity and positive predictive value for detection and correct classification of a given cystic lesion by TAUS with reference to final MRI reports. Additionally, we estimated inter-observer reliability adjusted for random agreement error between TAUS and MRI by calculating Cohen’s Kappa coefficient κ, using the free downloadable software R, version 3.6.3. The magnitude of κ was judged according to a categorical scale adapted from the foundational work by Landis and Koch [11] (Supplementary Table S1).

## 3. Results

### 3.1. Patient Characteristics

A total of 147 patients was analyzed, including 68 female and 79 male patients. The average age was 58 ± 15.4 years, ranging from 19 to 87 years. In total, 72 patients rated themselves as non-smokers, with 49 having never smoked in their lives. Smoking cessation was reported by another 23 patients. Thirty-four patients were active smokers, while 41 patients had not been inquired about their smoking habits. Eighty-one of patients reported moderate levels of alcohol consumption, while 15 and 13 admitted to former and ongoing alcohol abuse, respectively. In 38 patients, the amount of alcohol intake, if any, was unknown. Twenty-seven patients had been diagnosed with endocrine insufficiency of the pancreas. Sixteen patients were affected by exocrine insufficiency, with an average pancreatic elastase 1 of 83 µg/g stool. When interviewed on clinical symptoms, 65 patients complained of upper abdominal discomfort. A summary of all clinical features as described above is presented in Table 1.

### 3.2. Final Diagnosis Based on Discharge Summary vs. TAUS Findings

**Figure 1:**
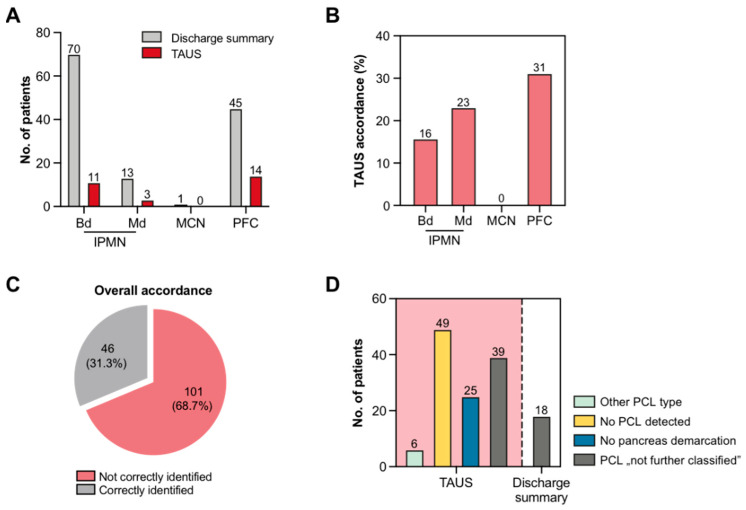
Diagnostic accordance between TAUS and final discharge summaries. (**A**) Frequency of different PCL entities as reported by TAUS and discharge summaries (in absolute numbers). (**B**) Frequency of different PCL entities as reported by TAUS (in percentage numbers). (**C**) Degree of overall accordance between TAUS and final discharge summaries. (**D**) TAUS-related causes of discrepant findings. Transabdominal ultrasound, TAUS; intraductal papillary mucinous neoplasm, IPMN; main duct type-IPMN, md-IPMN; branch duct type-IPMN, bd-IPMN; mucinous cystic neoplasm, MCN; post-pancreatitis fluid collection, PFC; pancreatic cystic lesion, PCL.

A major contribution to the definitive diagnosis as specified in the patient’s discharge summary was made by advanced imaging studies such as EUS and MRI, hence serving as a composite reference standard according to current guidelines [10]. Seventy patients were finally diagnosed with a bd-IPMN that was ascertained by TAUS in only 11 patients (Figure 1A,B). Another 13 patients received the diagnosis of md-IPMN, which was detected by TAUS in 3 patients. A single case of MCN was mentioned in the discharge summaries but was not discernible by TAUS. No SCN was found in the entire study population. A PFC was present in 45 patients, 14 of whom were correctly identified by TAUS. Overall accordance of TAUS with final discharge summaries was only 31%, while discrepancies were detected in 69% of patients (Figure 1C). Hereby, in most patients, no cyst could be detected by TAUS, albeit a clear visualization of the pancreas was obtained. In another 25 patients, the pancreas could not be demarcated by TAUS at all, while in 39 patients, PCLs were described as “not further classified” by TAUS. At the same time, a precise cyst classification was lacking in the discharge summaries of only 18 patients (Figure 1D).

### 3.3. TAUS vs. MRI

**Figure 2:**
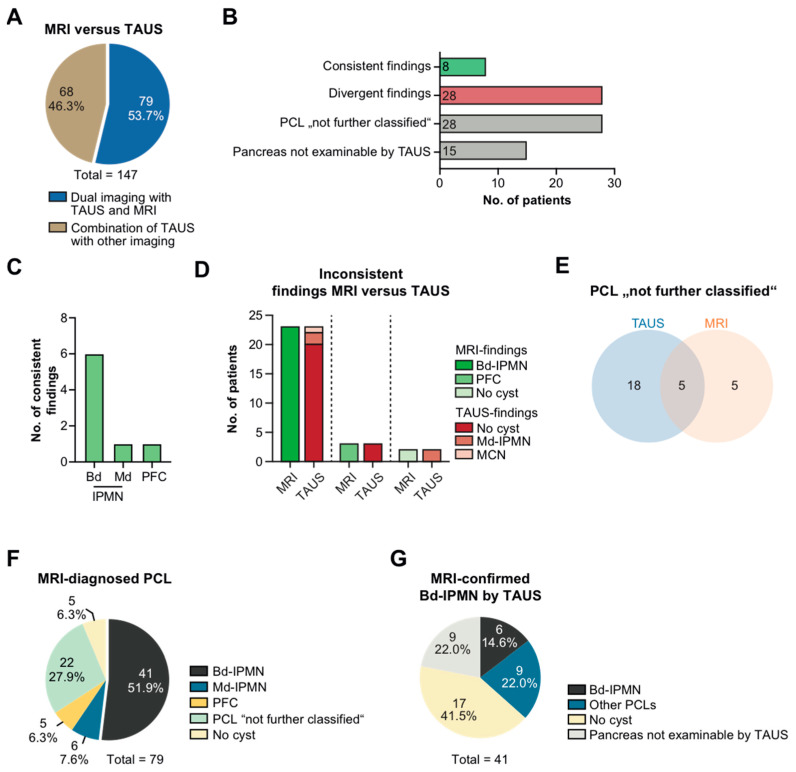
Diagnostic accordance between TAUS and MRI scans. (**A**) Patients with dual imaging combining TAUS with MRI or other type of imaging. (**B**) Patients (in absolute numbers) with MRI and TAUS assigned to the categories “consistent findings”, “divergent findings”, “PCL not further classified”, and “Pancreas not examinable by TAUS”. (**C**) Distribution of consistent findings. (**D**) Inconsistent findings between TAUS and MRI. (**E**) Venn diagram: non-classifiable PCL on TAUS and MRI. (**F**) Diagnostic categorization of PCLs evaluated by MRI (in absolute numbers). (**G**) Juxtaposition of MRI-confirmed bd-IPMN with TAUS-based assessment (in absolute numbers). Transabdominal ultrasound, TAUS; magnetic resonance imaging, MRI; intraductal papillary mucinous neoplasm, IPMN; main duct type-IPMN, md-IPMN; branch duct type-IPMN, bd-IPMN; mucinous cystic neoplasm, MCN; post-pancreatitis fluid collection, PFC; pancreatic cystic lesion, PCL.

Seventy-nine patients underwent dual imaging of the pancreas by TAUS and MRI (Figure 2A). In 8 patients, the findings obtained from both examinations were considered congruent, while they differed from one another in 28 patients. In 15 patients the pancreas could not be properly visualized by TAUS. A PCL was designated as “not further classified” in a total of 28 patients (Figure 2B). Consistent results largely originated from bd-IPMN, which were correctly identified by TAUS in only *n* = 6 patients (Figure 2C). Among aberrantly classified PCLs, the constellation of MRI-proven bd-IPMN that had been completely missed by TAUS was prevailing (Figure 2D). An indeterminate PCL occurred far more often with TAUS (*n* = 18 patients) than with MRI (*n* = 5 patients, Figure 2E). Based on MRI scans, bd-IPMN was the most commonly diagnosed PCL (Figure 2F). In 17 patients, TAUS failed to detect an actually existing bd-IPMN despite otherwise satisfactory visualization of the pancreas. In another 9 patients with MRI-confirmed bd-IPMN, the pancreas could not even be localized with TAUS (Figure 2G).

Inter-rater reliability was calculated for the total number of examinations (*n* = 79) and the number of examinations in which the pancreas was not or poorly visualized (*n* = 64), resulting in Cohen’s κ of 0.041 and 0.050, respectively (Supplementary Tables S2 and S3). If examinations were stratified for the investigators’ experience, ultrasound experts and basic-level trained ultrasonographers achieved a Cohen’s κ of 0.093 and 0.021, respectively. Although ultrasound experts trended towards a marginally better correlation with MRI-based ratings, κ scores altogether indicated a vanishing low level of inter-observer agreement, as corroborated by approximate significance values constantly exceeding 0.05 (Supplementary Table S3). Compared to MRI, sensitivity and the positive predictive value of detecting a PCL with TAUS were 54% and 86%, respectively (Supplementary Table S4). No remarkable change in these statistical values was observed when they were separately computed for the group of expert and basic-level trained TAUS investigators.

### 3.4. TAUS vs. EUS

**Figure 3:**
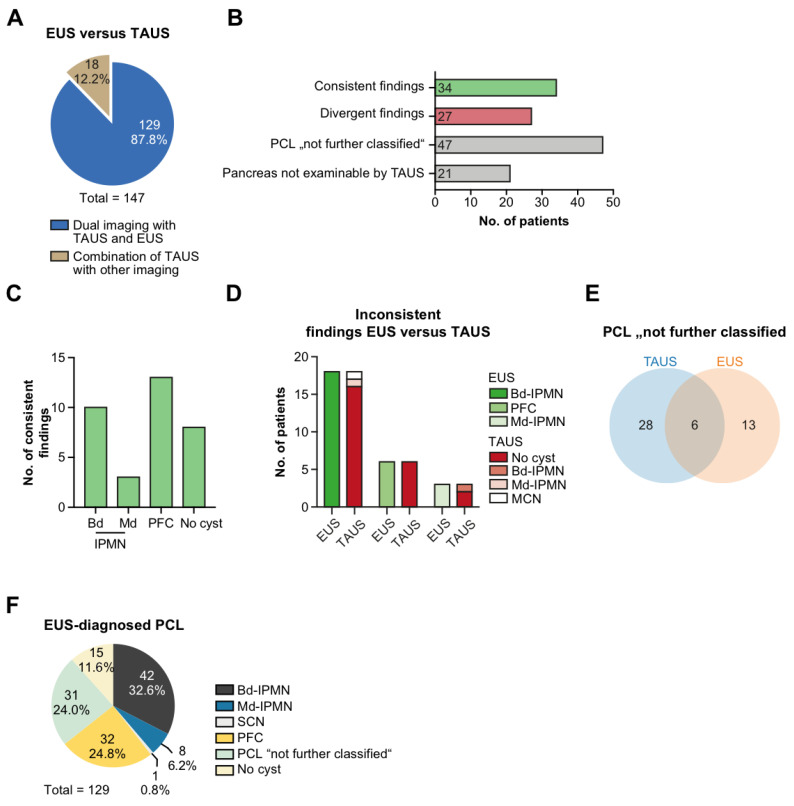
Diagnostic accordance between TAUS and EUS. (**A**) Patients with dual imaging combining TAUS with EUS or other type of imaging; (**B**) Patients (in absolute numbers) with EUS and TAUS assigned to the categories “consistent findings”, “divergent findings”, “PCL not further classified”, and “Pancreas not examinable by TAUS”. (**C**) Distribution of consistent findings. (**D**) Inconsistent findings between TAUS and EUS. (**E**) Venn diagram: non-classifiable PCLs on TAUS and EUS. (**F**) Diagnostic categorization of PCLs evaluated by EUS (in absolute numbers). Transabdominal ultrasound, TAUS; endoscopic ultrasound, EUS; intraductal papillary mucinous neoplasm, IPMN; main duct type-IPMN, md-IPMN; branch duct type-IPMN, bd-IPMN; mucinous cystic neoplasm, MCN; serous cystic neoplasm, SCN; post-pancreatitis fluid collection, PFC; pancreatic cystic lesion, PCL.

In 129 patients, TAUS examination was followed by a detailed exploration of the pancreas with EUS (Figure 3A). The findings were consistent in a total of 34 patients (Figure 3B). The PCL entities most likely allocated concordantly by TAUS and EUS were again PFC and bd-IPMN (*n* = 13 and *n* = 10, respectively; Figure 3B,C). On the other hand, discrepancies were noted in 27 patients which, for the most part, resulted from bd-IPMN and PFC detected by EUS but overlooked by TAUS (*n* = 16 and *n* = 6, respectively) despite an unobstructed view on the pancreas (Figure 3B,D). While no specific diagnosis could be established by EUS in 13 patients, TAUS remained inconclusive in approximately twice as many patients (Figure 3E). In 21 patients, the pancreas could not be inspected by TAUS, while it could always be visualized with EUS. The majority of PCLs diagnosed by EUS were related to bd-IPMN and PFC (*n* = 42 and *n* = 32, respectively; Figure 3F).

### 3.5. TAUS vs. CT

**Figure 4:**
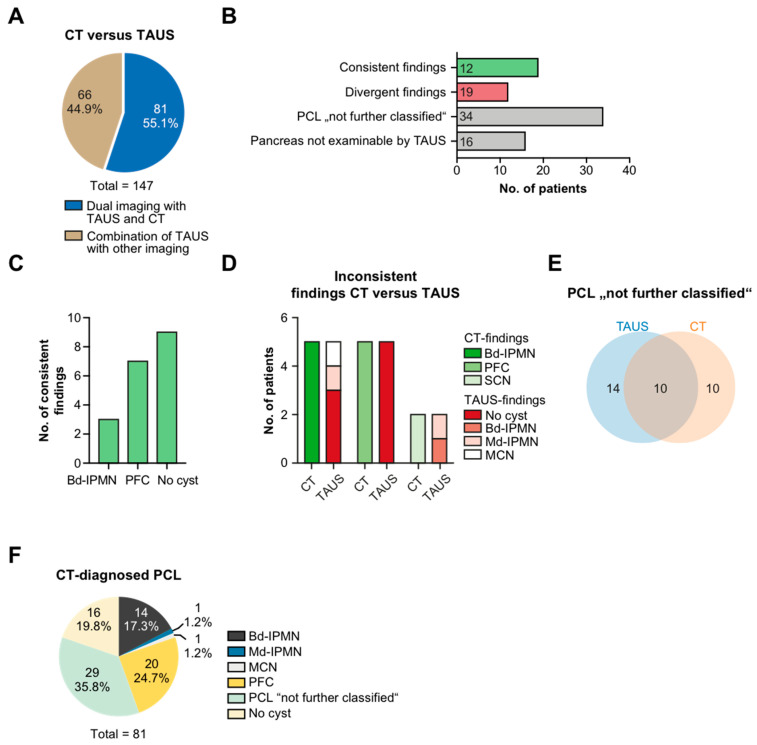
Diagnostic accordance between TAUS and CT scans. (**A**) Patients with dual imaging combining TAUS with CT or other type of imaging. (**B**) Patients (in absolute numbers) with CT and TAUS assigned to the categories “consistent findings”, “divergent findings”, “PCL not further classified”, and “Pancreas not examinable by TAUS”. (**C**) Distribution of consistent findings. (**D**) Inconsistent findings between TAUS and CT. (**E**) Venn diagram: non-classifiable PCLs on TAUS and CT. (**F**) Diagnostic categorization of PCL evaluated by CT (in absolute numbers). Transabdominal ultrasound, TAUS; computed tomography, CT; intraductal papillary mucinous neoplasm, IPMN; main duct type-IPMN, md-IPMN; branch duct type-IPMN, bd-IPMN; mucinous cystic neoplasm, MCN; serous cystic neoplasm, SCN; post-pancreatitis fluid collection, PFC; pancreatic cystic lesion, PCL.

A combined approach with TAUS and CT was adopted in 81 patients to search for PCLs (Figure 4A). The findings from both imaging modalities were found to be in accordance in 12 patients, while they differed in 19 patients. Visualization of the pancreas by TAUS was hampered in 16 patients, while it always succeeded with CT (Figure 4B).

Coincident reports most commonly pertained to PFC and patients in whom neither CT nor TAUS were able to locate a cystic lesion (Figure 4C). Inconsistent findings largely emerged from PFC and bd-IPMN, which were recognized by CT but not by TAUS (*n* = 5 and *n* = 3, respectively; Figure 4B,D). In a total of 34 patients, imaging was unable to disclose a definitive diagnosis yet was better with TAUS (*n* = 14) when compared with CT (*n* = 10) (Figure 4E). Furthermore, PCLs and PFCs represented the most frequent entities revealed by CT (*n* = 29 and *n* = 20, respectively; Figure 4F).

### 3.6. Number of Pancreatic Cysts

**Figure 5:**
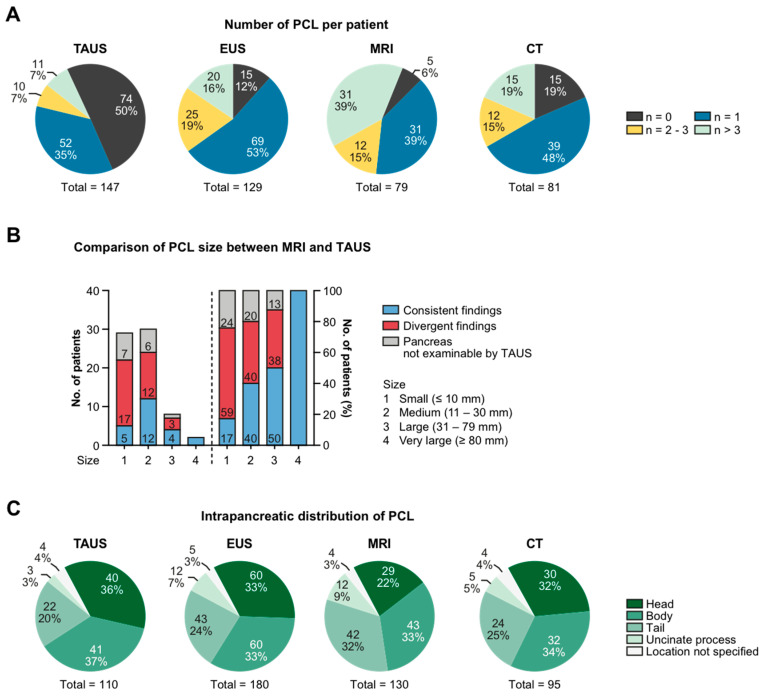
(**A**) Quantification of cysts per patient and imaging modality. (**B**) Comparison of PCL size determined by MRI with TAUS measurements; (**C**) Intrapancreatic localization of PCLs arranged by anatomical segment. Pancreatic cystic lesion, PCL; transabdominal ultrasound, TAUS; endoscopic ultrasound, EUS; magnetic resonance imaging, MRI; computed tomography, CT.

The number of pancreatic cysts per patient was counted separately for each type of imaging (Figure 5A). The finding of more than one cyst was collectively referred to as multiple cysts. TAUS was the imaging study most likely to completely miss existent cysts compared to EUS, MRI, and CT. Single cysts were most commonly recognized by EUS and CT, while for multiple cysts (*n* > 3), detection rates were highest with MRI.

### 3.7. Size of Pancreatic Cysts

Cysts were subdivided according to their size, which was determined by the maximum diameter of a given lesion (Figure 5B). Cysts measuring ≤10 mm were considered “small”, while cysts ranging from 11 to 30 mm and from 31 to 80 mm were considered “medium” and “large”, respectively. A cyst size exceeding 80 mm was defined as “very large”. Size quantification was acquired from MRI scans to ensure a precise classification of cysts. Based on these measurements, a permissible deviation of ±20% in cyst size was applied to the values from TAUS. The results from MRI and TAUS were regarded as “coinciding” if a cyst was allocated to the same metric category. Any cases in which TAUS, as opposed to MRI, either failed to report values or recorded discordant values, were rated as “differing”. In 5 patients, cysts were detected by MRI without, however, providing information on the size. In another 5 patients, TAUS examination yielded measurement data for cysts that could not be retrieved by MRI. These 10 patients were excluded from further analysis. Within the group of “small cysts”, only five PCLs were correctly assigned to this category, while for most lesions, the measurements differed between TAUS and MRI. In 7 patients, the pancreas could not be visualized. For medium-sized cysts, the numbers of coinciding and differing measurements were equal. In 6 patients, the pancreas was missed on TAUS scans. Half of all cysts judged as large on MRI were confirmed by TAUS, whereas the remaining PCLs were either misclassified (*n* = 3) or could not be detected (*n* = 1). Only for very large cysts, a 100% agreement between TAUS and MRI was reached. However, only two PCLs fell into this category.

### 3.8. Localization of Pancreatic Cysts

Several parts of the pancreas, including the head, uncinate process, body, and tail, were found to harbor cysts, featuring either solitary or multiple lesions in the same or different regions of the organ (Figure 5C). The highest burden of PCLs identified by TAUS was noted in the pancreatic head and body (*n* = 40 and *n* = 41, respectively) whereas detection probability sharply declined in the tail and the uncinate process (*n* = 22 and *n* = 3, respectively). By absolute numbers, EUS was characterized by superior resolution capacities for each part of the pancreas (head: *n* = 60; body: *n* = 60; tail: *n* = 43; uncinate process: *n* = 12), in part, even surpassing the spatial discrimination achieved by MRI (head: *n* = 29; body: *n* = 43; tail *n* = 42; uncinate process: *n* = 12). Compared with the aforementioned imaging modalities, CT scans yielded the lowest overall number of pancreatic cysts (head: *n* = 30; body: *n* = 32; tail *n* = 24; uncinate process: *n* = 5). However, referring to the total number of cysts per type of imaging, the percentage distribution of cysts among the different sections of the pancreas remained roughly the same.

### 3.9. Cyst Features

**Figure 6:**
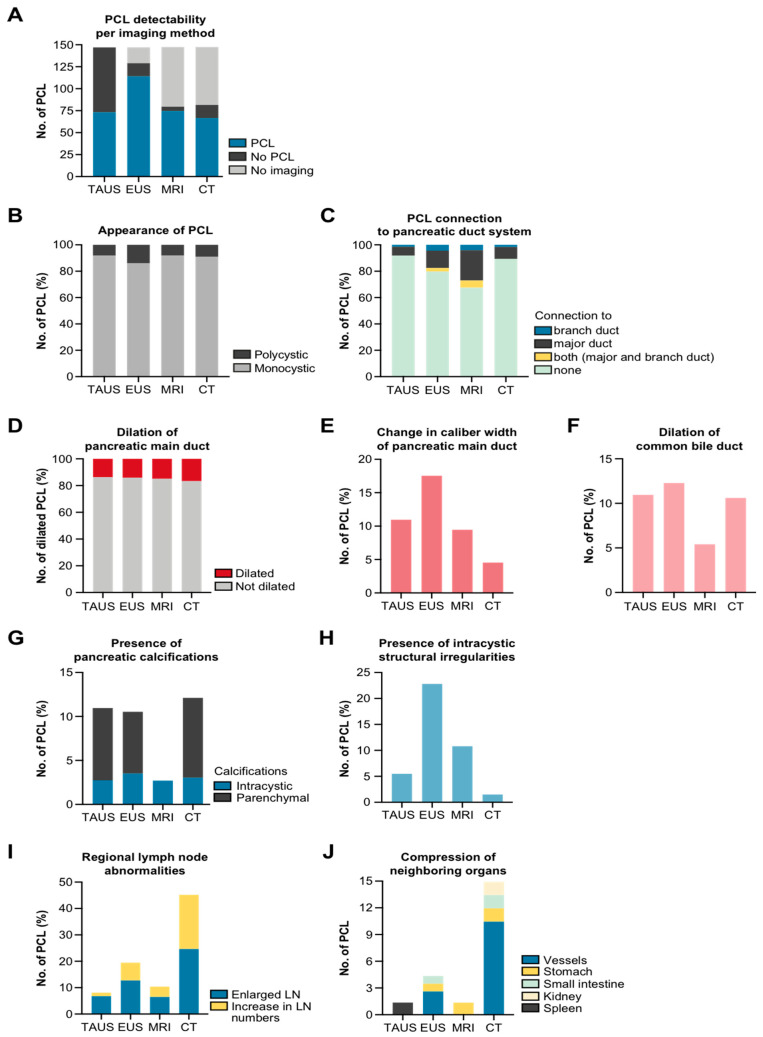
Discriminatory features to be used for detection of high-risk PCL. (**A**) TAUS examinations with at least one proven PCL per patient. (**B**) Architecture of PCL. (**C**) Connection of PCL to the pancreatic duct system. (**D**) Dilation of the pancreatic main duct. (**E**) Change in caliber width of the pancreatic main duct. (**F**) Dilation of the common bile duct. (**G**) Parenchymal and intracystic calcifications. (**H**) Irregular internal structuring of PCL. (**I**) Presence of regional lymphadenopathy. (**J**) Compression of neighboring organs by PCL. Pancreatic cystic lesion, PCL; transabdominal ultrasound, TAUS; endoscopic ultrasound, EUS; magnetic resonance imaging, MRI; computed tomography, CT.

Beyond the number, size, and localization of PCLs, classification systems rely on several additional features to help differentiate benign cysts from potentially malignant lesions. This section only includes examinations in which at least one pancreatic cyst had been ascertained by TAUS (*n* = 73 cysts), EUS (*n* = 114 cysts), MRI (*n* = 74 cysts), and CT (*n* = 66 cysts) (Figure 6A).

#### 3.9.1. Monocystic vs. Polycystic Configuration

The vast majority of PCLs exhibited a monocystic appearance and were detected to the same extent by all imaging methods. By contrast, only a very low number of lesions were classified as polycystic, amounting to a prevalence from 8 to 14% (Figure 6B).

#### 3.9.2. Connection to the Pancreatic Duct System

Each cyst was scrutinized for the existence of a connection to the pancreatic duct, differentiating between (1) lesions with a connection to the main duct, (2) with a connection to a branch duct, (3) with a connection to both the main and branch ducts, and (4) with no traceable connection (Figure 6C). Most pancreatic cysts were found to be devoid of a visible connection to the duct system. Among all available imaging methods, the prevalence of this type of cyst was particularly high when TAUS was used for investigation. To a lesser degree, the evaluations by EUS and MRI resulted in a cyst being classified as isolated. By contrast, cysts communicating with the main duct, a branch duct, or both were far more likely to be detected by EUS and MRI compared with TAUS and CT.

#### 3.9.3. Dilation and Changes in Width of the Main Pancreatic Duct and Common Bile Duct

In accordance with previous publications, the pancreatic main duct was considered significantly dilated if its diameter measured greater than 5 mm [12,13]. Normal-sized ducts were present in the majority of cases irrespective of the imaging modality employed. Conversely, a dilated duct was diagnosed in less than a fifth of cases, varying between 14% with CT and 17% with TAUS (Figure 6D).

Abrupt changes in duct width are considered an ominous finding that may either point to an underlying malignancy such as pancreatic cancer or may, itself, reflect a precursor lesion such as md-IPMN. The detectability of caliber changes was highest if EUS was used to examine the pancreas, while TAUS and MRI showed similar percentages (Figure 6E).

A dilated common bile duct (CBD) may signalize the presence of an obstructive mass lesion at the level of the pancreatic head or the ampullary region. Regardless of their biological behavior, pancreatic cysts may, at some point, substantially impede biliary drainage by impinging on the distal portion of the CBD. By consensus, a duct caliber of 4–6 mm is considered within normal range, while a CBD wider than 7 mm is defined as dilated [14,15]. Collectively, the proportion of patients with a concomitant CBD dilation was small, ranging between 5% and 12% (Figure 6F). No remarkable differences were noted upon comparison of TAUS with EUS and CT, respectively, while a dilated CBD was less frequently discovered on MRI scans.

#### 3.9.4. Calcifications and Internal Structure of Cysts

A variety of cystic lesions of the pancreas may contain calcifications, which however are not indicative of a specific pathological entity. In this study, calcified cysts were evaluated separately from diffuse parenchymal calcifications (Figure 6G). Across the entire spectrum of pancreas imaging, calcifications of any distribution pattern were absent in the vast majority of examinations. Expectably, the yield of parenchymal calcifications was highest on CT scans, while none were detected by MRI. The prevalence of parenchymal calcifications was nearly equal for TAUS and EUS. By contrast, the prevalence of calcified PCLs was even among all imaging modalities.

Unlike simple cysts, a number of pancreatic cysts exhibit distinct radiographic features, e.g., thickened walls, internal septation, nodules, and solid elements inside the lumen. Some, referred to as “worrisome features”, have found entrance into the international consensus Fukuoka guidelines, revised 2017, which are principally used to screen for IPMN and MCN with high-risk stigmata [16]. In this study, EUS, followed by MRI, proved to be the most sensitive method to dissect such complex cysts (Figure 6H).

#### 3.9.5. Regional Lymph Nodes and Compression of Adjacent Organs

A close inspection of the peripancreatic lymph nodes (LNs) of the pancreas is warranted in any case in which a pancreatic cyst is associated with worrisome features. Owing to its prognostic strength regarding progression of a pancreatic cyst to an invasive neoplasm, lymphadenopathy itself is listed as an independent “worrisome feature” [16]. In general, for most of the examinations, no LN abnormalities were reported regardless of the type of imaging applied. Enlarged LNs were most frequently encountered on CT scans, followed by EUS, MRI, and lastly TAUS. The imaging studies were ranked in the same order when the scans were analyzed for an increase in LN numbers (Figure 6I).

Rarely, pancreatic cysts were of a size large enough to exert a relevant space-occupying effect on the adjacent viscera and vasculature. Affected organs included the stomach, intestine, left kidney, spleen, and peripancreatic vessels. Investigations with TAUS and MRI yielded only one pancreatic cyst, causing displacement or impression of a neighboring organ (spleen and stomach, respectively). By contrast, several cases of relevant organ compression were detected by EUS and CT, for the most part involving local vessels (mesenteric artery/vein, splenic artery/vein, and portal vein) (Figure 6J).

## 4. Discussion and Conclusions

Over recent decades, pancreatic imaging has seen a remarkable uplift in quality of soft tissue contrast resolution and anatomical details, propelled by the advent of magnetic cross-sectional and EUS imaging modalities. In a prospective study, a surprisingly high baseline prevalence of 49.1% was reported for pancreatic cysts, with nearly two-thirds of all study subjects showing a progress in number and/or maximum cyst size within a 5-year follow-up period [17]. Already in 2018, the European Study Group on Cystic Tumours of the Pancreas published an evidence-based update of recommendations to help streamline the diagnostic evaluation and management of pancreatic cystic neoplasms [10].

While CT and MRI have an overall similar accuracy when characterizing a given PCL, CT is superior in detecting calcifications and typical tumor staging features. MRI with MRCP is particularly helpful to identify a connection of the PCL to the pancreatic ductal system and/or to detect septations or mural nodules. However, especially in the fine differentiation of worrisome features such as a contrast-enhancing mural nodules, EUS is recommended to provide complimentary information. An upgrade of native EUS to facilitate detection of the pancreatic microvasculature includes contrast-enhanced EUS with Power Doppler mode, which has been shown to improve sensitivity for the detection of small pancreatic carcinomas <2 cm with an irregular vascularization pattern [18]. This technology was further refined to process vascular signals with harmonic imaging, referred to as contrast harmonic endoscopic ultrasonography (CH-EUS) [19]. CH-EUS is nowadays recommended to delineate hyper-enhancing mural nodules, solid masses, or septations, suspicious of malignant transformation [10]. At the same time, guidelines concede that, even with the aid of EUS, the differentiation between benign and malignant PCLs continues to be challenging and, therefore, endorse cytological or histological validation in indeterminate cases. Compared to cross-sectional imaging with MRI and CT, EUS-FNA-guided acquisition of cyst fluid for quantification of carcinoembryonic antigen attained a significantly higher sensitivity for detecting high-grade dysplasias and pancreatic adenocarcinomas, respectively [20]. Diagnostic yield and accuracy of PCL sampling can be scaled up further by performing a through-the-needle biopsy (TTNB) of the cyst wall [21,22].

However, routine use of such invasive methods is barred by a non-negligible risk for major adverse events, especially with TTNB [23], limited infrastructural and personal resources, and cost-effectiveness considerations. For example, a basic TAUS costs approximately 40 €, while the expenses for endosonography of the upper gastrointestinal tract without biopsy and MRI amount to approximately 85 € and 500 €, respectively [24]. Therefore, foremost, due to its universal availability and absence of ionizing radiation, TAUS is still viewed as a central element in the diagnostic workflow of PCLs. In a prospective surveillance study, the likelihood of PCL visualization by TAUS was linked to patient sex, while it was inversely correlated with anterior–posterior abdominal diameter and weight [25]. Jeon et al. reported that pancreatic cysts incidentally discovered by TAUS were more likely to be present in younger and male patients [26]. However, in previous studies, the waist circumference did not significantly influence the detection rate. The same author group also stated that, if correlative imaging such as CT, MRI, and EUS had been performed prior to TAUS, the subsequent detection of pancreatic cysts by TAUS was significantly improved. The greatest gain in detectability was observed for cysts located in the uncinate process and for small cysts sized ≤25 mm. At the same time, the detection rates of cysts originating from the tail were barely enhanced [26]. These results confirm our finding that many cysts located in the uncinate process were potentially missed on primary TAUS but their detectability increased fourfold (*n* = 3 vs. *n* = 12) upon employing complementary imaging such as EUS or MRI. Additionally, the majority of cysts rated as small on MRI were either overlooked or falsely classified by TAUS (58.6%). Choi et al. could show that both a large size and location in the pancreatic neck or body facilitate detectability of focal pancreatic lesions by TAUS [27]. These findings are in line with two other studies, indicating a significantly higher detection rate of pancreatic cysts located outside the tail and cysts ≥10 mm [28,29]. Indeed, we were able to demonstrate that, in most cases, cysts graded as large or very large (i.e., measuring >30 mm and >80 mm, respectively) were reliably localized by TAUS and matched the size measured by MRI. Conceivably, due to their relatively short distance to the anterior abdominal wall, the head and body are the most exposed parts of the pancreas, predicting higher chances of visualization by TAUS. However, in our study, the majority of cysts occurred in the pancreatic head and body, irrespective of whether TAUS or other more sensitive imaging such as EUS or MRI were performed.

Based on further results from the previously cited study, it becomes evident that the multiplicity of pancreatic cysts also impacts detection rates of TAUS, which proved inferior for multiple cysts compared to single cysts [28]. In our study, the percentage of patients with single cysts identified by TAUS amounted to 71.2%, while multiple cysts (>3) were completely captured in only 15% of patients. At the same time, TAUS was sufficient to accurately discriminate between a monocystic and a polycystic configuration.

A shortcoming of TAUS refers to its poor ability to trace a connection between a cystic lesion and the ductal system, which is of paramount importance to discriminate between md-IPMN, bd-IPMN, and m-IPMN and to estimate their respective potential for malignant degeneration. Our data confirm the observation that successfully uncovering an existing communication between a cyst and the duct by TAUS alone is a rarity and is more faithfully accomplished by MRI or EUS [30,31]. Additionally, structural inhomogeneities within cysts such as thickened walls, internal septa, nodules, and solid elements were most reliably distinguished by EUS followed by MRI, which is consistent with studies by Du et al. and Li et al. [32,33]. On the other hand, pathological dilations of the CBD and the pancreatic main duct were identified to the same extent by TAUS as by other imaging modalities.

We were unable to find studies specifically examining the impact of the individual investigator’s skills on the quality of TAUS imaging. Indeed, we found that ultrasound experts marginally outperformed basic-level trained investigators, attaining a slightly higher index of agreement with MRI imaging. However, κ values falling within a range below 0.2 are to be considered incommensurate to clarify a PCL by TAUS alone.

Contrary to valid guidelines issued by international specialist societies [9,10], a few institutions have incorporated TAUS into their regular surveillance program for patients with PCLs. An MRI-restrictive follow-up applied to patients with “low risk” PCLs without indication for prompt surgical intervention yielded promising results, suggesting that TAUS could offer a safe—and at the same time cost-saving—surveillance approach provided that the patient population is carefully chosen [34,35]. By contrast, a hypothetical cost-effectiveness analysis revealed that guideline-adherent surveillance of incidental pancreatic cysts proved to be an unfavorable strategy compared to upfront surgery unless diagnostic specificity was dramatically improved, thereby preventing resection of low-risk PCLs [36]. It is therefore questionable whether the cost-saving effect of TAUS could generally compensate for its lack of diagnostic precision and specificity. This becomes particularly relevant in Germany, where costs for the guideline-recommended EUS are not that much higher when compared to a basic TAUS (40 vs. 85 €) [24]. Nevertheless, given the relentless economic pressure, steps should be undertaken to define the circumstances under which TAUS could safely take precedence (e.g., size, location, and low-grade appearance of PCLs, body mass index/abdominal girth, and age of a patient).

Although the significance of this study is impaired by its retrospective design and overall small patient numbers, the following conclusions can be drawn: In our view, owing to its comparably poor detection rates, especially for PCLs of concern such as IPMN and low inter-rater reliability, TAUS does not qualify as a standalone imaging modality and should always be flanked by an MRI scan or equivalent imaging, confirming valid guidelines. That being said, we think that the results of this study should not discount the enormous economic advantages of TAUS but instead serve as a starting point to opening up new fields of application. If TAUS is intended for use as an adjunct monitoring tool, both morphological characteristics of the PCL such as size and location, and individual patient factors should be taken into consideration to select suitable candidates and thus to minimize misinterpretation of a given PCL. Furthermore, ultrasound investigators ought to have similar records of hands-on experience in order to ensure constant quality levels of TAUS examinations. Taken together, a prudent use of TAUS, either for screening in a two-tier system or for follow-up of a known PCL, may provide novel information and help economize costly and elaborate imaging.

## Figures and Tables

**Table 1:** Overview of central clinical elements pertinent to chronic pancreatic disease.

**Sex**	**No. of patients (%)**
Male	79 (54%)
Female	68 (46%)
**Age**	**Mean/range (years)**
	58/19–87
**Smoking habits**	**No. of patients (%)**
Never smoked	49 (33%)
Former smoking	23 (16%)
Active smoking	34 (23%)
Not known	41 (28%)
**Consumption of alcoholic beverages**	**No. of patients (%)**
Never	81 (55%)
Former alcohol intake	15 (10%)
Continued alcohol intake	13 (9%)
Not known	38 (26%)
**Epigastric discomfort**	**No. of patients (%)**
Yes	65 (44%)
No	82 (56%)
**Endocrine insufficiency**	**No. of patients (%)**
Yes	27 (18%)
No	120 (82%)
**Exocrine insufficiency**	**No. of patients (%)**
Yes	16 (11%)
No	131 (89%)

## Data Availability

Not applicable.

## Supplementary Materials

The following supporting information can be downloaded at: https://www.mdpi.com/article/10.3390/jcm11206176/s1, Table S1: Interpretation of Cohen’s Kappa coefficient κ as proposed by Landis and Koch [11]; Table S2: The crosstabulation displays all cases including those patients in whom the pancreas could not be visualized by TAUS; Table S3: The crosstabulation displays all cases excluding those patients in whom the pancreas could not be visualized by TAUS. Besides the entirety of TAUS investigators, inter-modal agreement between TAUS and MRI was separately calculated within the group of experienced and basic-level trained investigators; Table S4: Illustration of deriving sensitivity and positive predictive value of TAUS-based PCL detection compared to MRI results. All point estimates are reported with their 95 % confidence intervals. Calculation of metrics was further stratified for the training level of investigators (a) experienced investigators, (b) basic-level trained investigators.
