# Supplementary material for: A Systematic Comparative Study on the Diagnostic Value of Transabdominal Ultrasound in Patients with Pancreatic Cystic Lesions
Source: J Clin Med. 2022 Oct 19;11(20):6176. doi: 10.3390/jcm11206176 (PMC9605155; doi:10.3390/jcm11206176)
Supplement: Supplementary file 1 [file jcm-11-06176-s001.zip › jcm-1894502-supplementary.pdf]

**Value of κ**	**Level of agreement**
< 0	No agreement
0 - 0.20	None to slight agreement
0.21 - 0.40	Fair agreement
0.41 - 0.60	Moderate agreement
0.61 - 0.80	Substantial agreement
0.81 - 1.00	Almost perfect agreement

Table S1: Interpretation of Cohen's Kappa coefficient κ as proposed by Landis and Koch [11].

**TAUS x MRI Crosstabulation**								
Count								
		MRI						Total
		1	2	3	5	6	7	
TAUS	0	9	2	0	1	0	3	15
	1	6	1	0	0	0	0	7
	2	2	1	0	0	2	0	5
	3	1	0	0	0	0	0	1
	5	0	0	0	1	0	0	1
	6	19	0	0	3	0	5	27
	7	10	3	2	0	3	5	23
Total		47	7	2	5	5	13	79

**Symmetric measures**					
		Value	Asymptotic Standard Error	Approximate T	Approximate significance
Measure of agreement	Kappa	0,041	0,038	1,132	0,258
N of valid cases		79			

Table S2: The crosstabulation displays all cases including those patients in whom the pancreas could not be visualized by TAUS.

TAUS x MRI Crosstabulation								
Count								
Experienced investigators		**MRI**						Total
		1	2	3	5	6	7	
TAUS	1	4		0	0	0	0	4
	2	2		0	0	2	0	4
	3	1		0	0	0	0	1
	5	0		0	1	0	0	1
	6	8		0	1	0	2	11
	7	3		1	0	1	2	7
Total		18		1	2	3	4	28
Basic-level trained investigators		MRI						Total
		1	2	3	5	6	7	
TAUS	1	2	1	0	0	0	0	3
	2	0	1	0	0	0	0	1
	6	11	0	0	2	0	3	16
	7	7	3	1	0	2	3	16
Total		20	5	1	2	2	6	36
All investigators		**MRI**						Total
		1	2	3	5	6	7	
TAUS	1	6	1	0	0	0	0	7
	2	2	1	0	0	2	0	5
	3	1	0	0	0	0	0	1
	5	0	0	0	1	0	0	1
	6	19	0	0	3	0	5	27
	7	10	3	2	0	3	5	23

Total	38	5	2	4	5	10	64

	Symmetric measures					
			Value	Asymptotic Standard Error	Approximate T	Approximate significance
Experienced investigators	Measure of agreement	Kappa	0,093	0,078	1,307	0,191
	N of valid cases		28			
Basic-level trained investigators	Measure of agreement	Kappa	0,021	0,060	0,369	0,712
	N of valid cases		36			
All investigators	Measure of agreement	Kappa	0,050	0,049	1,074	0,283
	N of valid cases		64			

Table S3: The crosstabulation displays all cases excluding those patients in whom the pancreas could not be visualized by TAUS. Besides the entirety of TAUS investigators, inter-modal agreement between TAUS and MRI was separately calculated within the group of experienced and basic-level trained investigators.

**All investigators**	PCL detectable on MRI	PCL not detectable on MRI	Total
PCL detectable on TAUS	32	5	37
PCL not detectable on TAUS	27	0	27
	59	5	64

Sensitivity: 0.54 (0.41, 0.67)
Specificity: 0.00 (0.00, 0.52)
Positive predictive value: 0.86 (0.71, 0.95)
Negative predictive value: 0.00 (0.00, 0.13)

**Experienced investigators**	PCL detectable on MRI	PCL not detectable on MRI	Total
PCL detectable on TAUS	14	3	17
PCL not detectable on TAUS	11	0	11

	25	3	28

Sensitivity: 0.56 (0.35, 0.76)
Specificity: 0.00 (0.00, 0.71)
Positive predictive value: 0.82 (0.57, 0.96)
Negative predictive value: 0.00 (0.00, 0.28)

**Basic-level trained investigators**	PCL detectable on MRI	PCL not detectable on MRI	Total
PCL detectable on TAUS	18	2	20
PCL not detectable on TAUS	16	0	16
	34	2	36

Sensitivity: 0.53 (0.35, 0.70)
Specificity: 0.00 (0.00, 0.84)
Positive predictive value: 0.90 (0.68, 0.99)
Negative predictive value: 0.00 (0.00, 0.21)

Table S4: Illustration of deriving sensitivity and positive predictive value of TAUS-based PCL detection compared to MRI results. All point estimates are reported with their 95 % confidence intervals. Calculation of metrics was further stratified for the training level of investigators (**a)** experienced investigators, **b)** basic-level trained investigators.
